# Spatiotemporal trends and determinants of syphilis among heterosexual males and females in the Netherlands, 2011 to 2023

**DOI:** 10.2807/1560-7917.ES.2025.30.49.2500364

**Published:** 2025-12-11

**Authors:** Laura Kayaert, Carolina JG Kampman, Helena CM Driessen-Hulshof, Birgit HB van Benthem

**Affiliations:** 1 Centre for Infectious Disease Control, National Institute for Public Health and the Environment (RIVM), Bilthoven, the Netherlands; 2 Public Health Service Twente (GGD Twente), Enschede, the Netherlands; 3 Public Health Service Zuid Limburg (GGD Zuid Limburg), Heerlen, the Netherlands

**Keywords:** syphilis, lues, STI, sexually transmitted infection, Netherlands, females, heterosexual males, heterosexuals

## Abstract

**BACKGROUND:**

Syphilis is increasing among females and heterosexual males (heterosexuals) in Europe and other Western countries. In the Netherlands, there has been an increase in syphilis positivity and diagnoses over the last 10 years. Research on syphilis among heterosexuals and on spatiotemporal syphilis trends in the Netherlands is limited.

**AIM:**

We aimed to assess spatiotemporal trends and identify determinants of syphilis among heterosexuals in the Netherlands.

**METHODS:**

This study used national surveillance data of all sexual health centres (SHCs) and included consultations among heterosexuals tested for syphilis between 2011 and 2023. Syphilis was defined as an infectious syphilis diagnosis (primary, secondary or early latent). Spatiotemporal trends were assessed using SaTScan, adjusting for demographic and behavioural factors. We performed a multivariate logistic regression to identify demographic and behavioural determinants of syphilis.

**RESULTS:**

We analysed 694,698 STI consultations among heterosexuals and identified 686 syphilis diagnoses. We found an increase in syphilis positivity from 0.05% in 2011 to 0.23% in 2023. SaTScan identified two space-time clusters; one in the north (2020–23) and one in the south-west (2019–23) of the Netherlands. Living with HIV (adjusted odds ratio (aOR): 17.48; 95% CI: 11.75–25.08) and having symptoms (aOR: 2.73; 95% CI: 2.34–3.20) were most strongly associated with syphilis.

**CONCLUSION:**

We found an increasing trend of syphilis among heterosexuals diagnosed at SHCs in the Netherlands. Syphilis was found in all regions. Since living with HIV was the strongest risk factor for syphilis, increased testing among heterosexuals living with HIV, both at SHCs and HIV treatment clinics, is recommended.

**Key public health message**

**What did you want to address in this study and why?**

Syphilis is a sexually transmitted infection that can cause serious health complications. The number of syphilis infections has been rising among females and heterosexual males (heterosexuals) in the Netherlands in the last decade. We set out to assess whether there were certain regions in the country with more syphilis than others and what the risk factors are for syphilis among heterosexuals.

**What have we learnt from this study?**

Our findings confirm that syphilis is increasing among heterosexuals in the Netherlands, from 0.05% in 2011 to 0.23% in 2023. We also found that syphilis is present in all regions, and that heterosexuals living with HIV have an increased risk of syphilis.

**What are the implications of your findings for public health?**

We found that current Dutch syphilis testing criteria successfully target the majority of people who have an increased risk of syphilis, but could be expanded further to reach everyone at risk. We recommend more focus on syphilis testing among heterosexuals living with HIV, who are currently not included in the testing criteria, so that this group can get a timely diagnosis and treatment.

## Introduction

Syphilis, a sexually transmitted disease (STI) caused by the bacterium *Treponema pallidum*, can cause serious health complications if not treated timely, such as neurological and cardiovascular problems. During the primary, secondary and early latent phases, individuals can infect others through sexual or blood contact [1]. An untreated infectious syphilis infection during pregnancy can be transmitted in utero from mother to child [2,3].

Adult syphilis is considerably less common in heterosexual males or females than in men who have sex with men (MSM). However, the number of cases in the European Union/European Economic Area (EU/EEA) increased by 29% among heterosexual males and by 32% among females between 2018 and 2022. The notification rate for females increased from 1.4 to 1.7 cases per 100,000 population in the same time period [4]. Possibly related to this, the congenital syphilis notification rate increased from 2.0 in 2018 to 2.4 per 100,000 live births in 2022 [5]. The increase in syphilis among heterosexuals could be linked to the increase among MSM. Bridging, which is the transmission of STIs across different sexual networks, has previously been found for bacterial STIs, including syphilis, in other countries [6-8]. Previous studies have found an increased risk of syphilis among heterosexuals with lower socioeconomic status, lower education, using drugs and working as a sex worker [9,10].

In the Netherlands, the number of infectious syphilis (primary, secondary or early latent, hereafter referred to as syphilis) diagnoses among females and heterosexual males is low compared with MSM, but the test positivity at sexual health centres (SHCs) increased from 0.14% to 0.35% for heterosexual males and from 0.07% to 0.17% for females between 2018 and 2023. The yearly number of congenital syphilis cases has been between zero and three in the past 10 years [11]. Given the lack of studies on syphilis among heterosexuals in the Netherlands, it is unknown whether the rates are equal across the Netherlands or whether some regions have more syphilis than others. Spatiotemporal tools provide information on space-time trends and have previously been used to assess the distribution of congenital syphilis in other countries [12,13]. 

In the current study, we aimed to assess spatial and temporal trends and identify determinants of syphilis among heterosexual males and females attending SHCs in the Netherlands. This will provide more knowledge on where in the country and in which key populations syphilis occurs, so that prevention and testing interventions can be more targeted.

## Methods

### Study setting and population

Syphilis is not a mandatory notifiable disease in the Netherlands. There are four main sources of syphilis data in the Netherlands: sexual health centres (SHCs), general practices (GPs), pregnancy screening, and commercial testing providers. Data from SHCs serve as the primary source for monitoring syphilis trends. There are 25 health regions in the country, of which 24 have an SHC, which can have multiple locations within their region. The SHCs provide free-of-charge, anonymous STI testing for people with an increased risk of STI.

Clients can book an STI consultation if they fulfil one or more criteria for testing, such as being younger than 25 years old, having STI symptoms, having received a partner notification or working as a sex worker. Until 2014, SHCs tested all heterosexual clients for syphilis. From 2015 onwards, only heterosexual clients who fulfil one or more of the syphilis-specific testing criteria received a syphilis test: 25 years or older, have been notified by a partner, have symptoms of syphilis, be a migrant from a region included in triage and be testing for the first time, have a partner who is MSM or from a region included in triage, have experienced sexual violence, or do sex work or have a partner who does sex work [14]. Demographic, behavioural, testing and diagnostic information is registered in the SHC’s electronic patient system for every consultation and compiled in a national database. From mid-2014 onwards, a person identifier is available for each consultation.

We selected data from all 24 SHCs in the Netherlands between 2011 and 2023. We included all consultations with a syphilis test among females and heterosexual males (hereafter collectively referred to as ‘heterosexuals’). Females were defined as cisgender women. Females who reported only female partners or both male and female partners were also included (9.6% of all consultations among females). Heterosexual males were defined as cisgender men who reported only having sex with women and/or other persons with a vagina. Heterosexual males who reported being MSM in other consultations were included (4.1% of all consultations with a person identifier among males).

### Variables

A syphilis diagnosis was defined as an infectious syphilis diagnosis (primary, secondary, or early latent syphilis) based on laboratory results. At the SHCs, syphilis is diagnosed by hemagglutination/agglutination assay (TPHA/TP-PA) or enzyme immunoassay (TP-EIA), followed by a fluorescent treponemal antibody absorption (FTA-ABS) test or Western blot. If positive, a rapid plasma reagin (RPR) is conducted to determine the activity of the infection [15]. 

Years were categorised based on changes in SHC testing policy; during 2011–14, SHCs tested all heterosexual clients for syphilis. From 2015 onwards, only heterosexual clients 25 years or older and those who fulfil one of the requirements as outlined above were tested for syphilis [14]. During 2020 and 2021, SHCs reduced testing substantially and applied stricter triage criteria because of the COVID-19 pandemic, which resulted in fewer consultations and prioritisation of those with high risk of STI.

Demographic variables included sex, age (categorised as ≤ 25 or > 25 years) and migration status. Migration status was determined by the client’s country of birth, and categorised as: born in the Netherlands, Western migrant, non-Western migrant, and unknown. The category Western migrant included persons born in northern, western (excluding the Netherlands), southern, or central Europe, North America or Oceania. The category non-Western migrant included migrants from all other countries.

Symptoms were self-reported and included all symptoms that can indicate an STI. Partner notification was defined as having received a notification from a (past) sexual partner for any STI. Sex work and the number of sexual partners were self-reported and pertained to the 6 months before the consultation. The number of partners was categorised in 0–1, 2–3, 4–5, ≥ 6 and unknown. HIV status was coded as positive if the client reported having had a positive HIV test in the past or if they received an HIV diagnosis in the same consultation. All other individuals were coded as HIV negative.

### Statistical analyses

For consultations where a person identifier was available, we assessed the number of syphilis tests and diagnoses per person during the study period. Additionally, we assessed whether the syphilis diagnosis occurred in the first or a subsequent consultation.

We calculated syphilis positivity by year (percentage of consultations with a syphilis diagnosis), for the total population and for males and females separately. A linear regression was performed to assess the trend over time for the total population, and another with an interaction term between year and sex to assess the difference between males and females.

We used the global Moran’s I statistic to assess global spatial autocorrelation. The input was the syphilis positivity per SHC region, aggregated across all years. SaTScan (https://www.satscan.org) was used to assess spatiotemporal trends in the years 2011–23, using the SHC regions as spatial units. SaTScan uses case and population data to detect non-random space-time clusters. The analysis was performed with a spatial window of 50% and 999 Monte Carlo replications; a Poisson distribution of the cases was assumed. The outcome was syphilis diagnosis, and the denominator was syphilis tests. We adjusted for sex, age, symptoms, partner notification, migration status, number of partners and HIV status.

Univariate logistic regressions were performed for each independent variable to assess their associations with syphilis. Variables with a p value < 0.2 were included in the multivariate logistic regression model. A p value < 0.05 was considered statistically significant in the multivariate model.

We performed three sensitivity analyses. Firstly, as testing criteria differed between individuals aged 25 or younger and individuals older than 25, we ran the multivariate model stratified by age group. Secondly, information on whether females had an MSM partner was only available from 2016 onwards, thus we ran another multivariate model for years 2016–23 including this potential factor. Thirdly, because information on partner notification for syphilis (as opposed to general STI partner notification) was only available from 2015 onwards, we performed a separate multivariate analysis for years 2015–23 including this potential factor.

Spatiotemporal analysis was conducted using the SaTScan software version 9.4.1. All data cleaning, other statistical analyses, and map creation were done using R version 4.4.1.

## Results

### Study population

We identified 694,698 STI consultations with a syphilis test among heterosexuals between 2011 and 2023. Of all consultations, 38.9% (n = 270,378 were among males, 61.1% (n = 424,320) were among females and 46.5% (n = 323,311) were among people older than 25 years. In 38.6% of consultations, individuals reported having STI symptoms, and in 20.9% of consultations, they reported having received a partner notification for an STI. Of all consultations, 0.2% were among persons living with HIV (Table). There were 686 consultations with a syphilis diagnosis. Of the 31 persons living with HIV who had a syphilis diagnosis, eight got diagnosed with HIV in the same consultation as the syphilis diagnosis and 22 were known HIV-positive.

**Table:** Factors associated with syphilis diagnoses, on consultation level, among females and heterosexual males tested for syphilis at sexual health centres in the Netherlands, 2011–2023 (n = 694,698)

Characteristics	Consultation without syphilis diagnosis n = 694,012		Consultation with syphilis diagnosis n = 686		Univariate analysis			Multivariate analysis		
	n	%	n	%	OR	95% CI	p value	aOR^a^	95% CI	p value
**Years**										
2011–2014	351,837	50.7	196	28.6	Reference			Reference		
2015–2019	221,190	31.9	235	34.3	1.91	1.58–2.31	< 0.001	1.69	1.40–2.05	< 0.001
2020–2021	51,593	7.4	106	15.5	3.69	2.90–4.66	< 0.001	3.34	2.62–4.23	< 0.001
2022–2023	69,392	10.0	149	21.7	3.85	3.11–4.77	< 0.001	3.63	2.92–4.50	< 0.001
**Sex**										
Female	424,041	61.1	279	40.7	Reference			Reference		
Male	269,971	38.9	407	59.3	2.29	1.97–2.67	< 0.001	2.24	1.91–2.62	< 0.001
**Age group**										
≤ 25 years	371,139	53.5	195	2.4	Reference			Reference		
> 25 years	322,820	46.5	491	71.6	2.89	2.46–3.42	< 0.001	2.37	2.00–2.81	< 0.001
Missing	53	NA	0	NA	NA			NA		
**Symptoms**										
No symptoms	422,788	60.9	260	37.9	Reference			Reference		
Symptoms	267,757	38.6	424	61.8	2.57	2.21–3.01	< 0.001	2.73	2.34–3.20	< 0.001
Unknown	3,467	0.5	2	0.3	0.94	0.16–2.92	> 0.9	0.60	0.10–1.94	0.5
**Partner notification**										
None	547,818	78.9	425	62.0	Reference			Reference		
Received notification	144,961	20.9	259	37.8	2.30	1.97–2.69	< 0.001	2.15	1.83–2.52	< 0.001
Unknown	1,233	0.2	2	0.3	2.09	0.35–6.49	0.3	2.22	0.35–7.44	0.3
**Sex work in past 6 months^b^**										
None reported	626,459	90.3	617	89.9	Reference			NA		
Reported sex work	65,522	9.4	59	8.6	0.91	0.69–1.18	0.5	NA		
Unknown	2,031	0.3	10	1.5	5.00	2.49–8.84	< 0.001	NA		
**Migration status**										
Born in the Netherlands	527,661	76.0	489	71.3	Reference			Reference		
Western migrant	27,556	4.0	15	2.2	0.59	0.34–0.95	0.042	0.50	0.29–0.81	0.008
Non-Western migrant	129,047	18.6	181	26.4	1.51	1.27–1.79	< 0.001	1.22	1.02–1.45	0.028
Unknown	9,748	1.4	1	0.1	0.11	0.01–0.49	0.028	0.16	0.01–0.73	0.072
**Number of sexual partners in past 6 months**										
0–1	152,529	22.0	261	38.0	Reference			Reference		
2–3	264,670	38.1	213	31.0	0.47	0.39–0.56	< 0.001	0.46	0.39–0.56	< 0.001
4–5	116,642	16.8	76	11.1	0.38	0.29–0.49	< 0.001	0.36	0.28–0.47	< 0.001
≥ 6	131,690	19.0	112	16.3	0.50	0.40–0.62	< 0.001	0.45	0.36–0.57	< 0.001
Unknown	28,481	4.1	24	3.5	0.49	0.32–0.73	< 0.001	0.77	0.49–1.16	0.2
**HIV status**										
Negative	692,776	99.8	655	95.5	Reference			Reference		
Positive	1,236	0.2	31	4.5	26.53	18.05–37.51	< 0.001	17.48	11.75–25.08	< 0.001

aOR: adjusted odds ratio; CI: confidence interval; NA: not applicable; OR: odds ratio; ref: reference level.

^a^ Factors included in multivariate model: years, sex, age group, symptoms, partner notification, migration status, number of sexual partners in the past 6 months and HIV status.

^b^ Excluded from multivariate model.

For the time period that a person identifier was available (mid-2014–23), 403,625 consultations could be linked to an individual. There were 268,330 unique individuals, of whom 75% (n = 200,837) had only one consultation. There were no individuals with more than one syphilis diagnosis. Of 528 syphilis diagnoses with a person identifier, 441 (84%) were in the first consultation of that person. Of these 441 diagnoses in the first consultation, 110 (25%) were among non-Western migrants.

### Temporal and spatiotemporal trends

**Figure 1:**
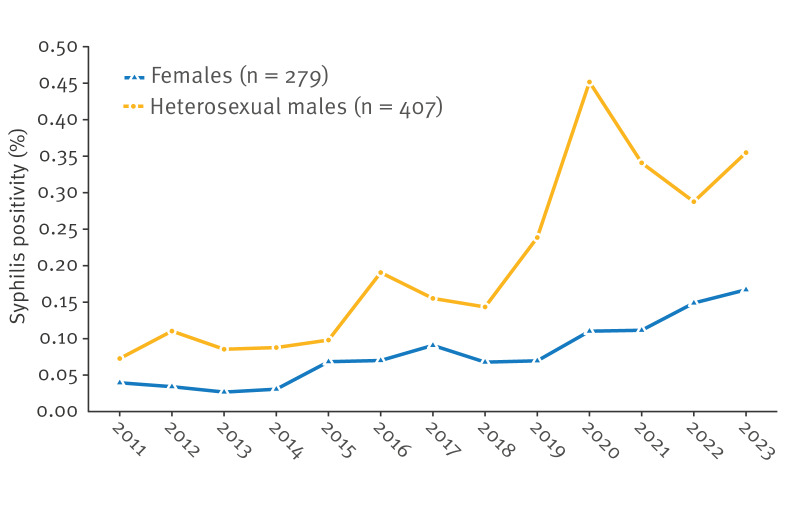
Trend in syphilis positivity among females and heterosexual males tested for syphilis at sexual health clinics, the Netherlands, 2011–2023 (n = 686)

There was an increase in the number of syphilis diagnoses between 2011 and 2023, from 48 in 2011 to 81 in 2023. The syphilis positivity increased from 0.05% to 0.23% in the same time period (Figure 1). The linear regression analysis showed that the total syphilis positivity increased by 0.016 for each year (p value < 0.001). The model including the interaction term showed that the increase in syphilis positivity over time was greater for males than for females (p value = 0.004).

**Figure 2:**
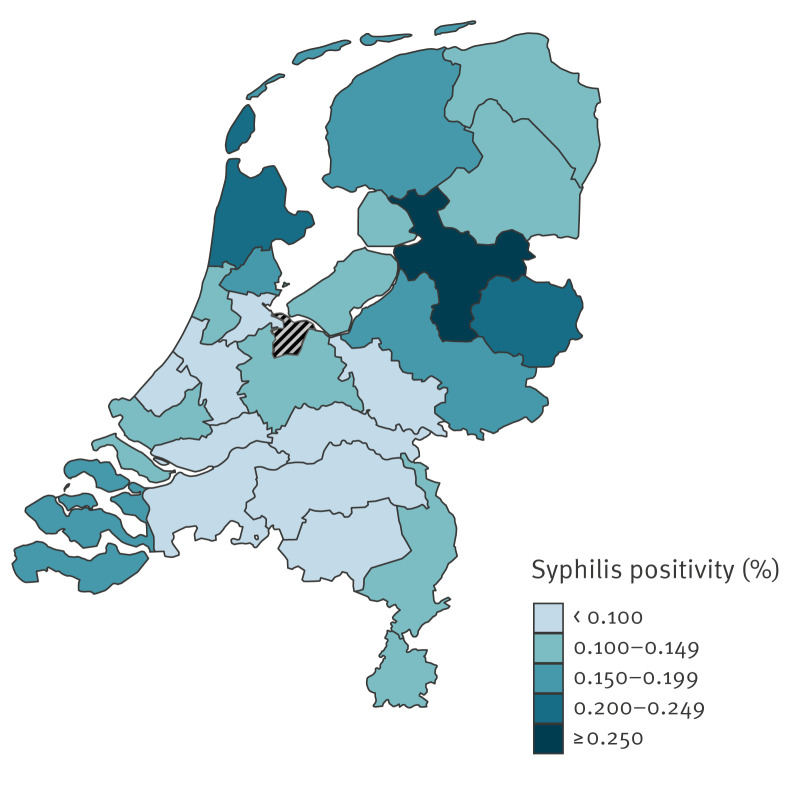
Aggregated syphilis positivity among females and heterosexual males per sexual health centre region in the Netherlands, 2011–2023 (n = 686)

**Figure 3:**
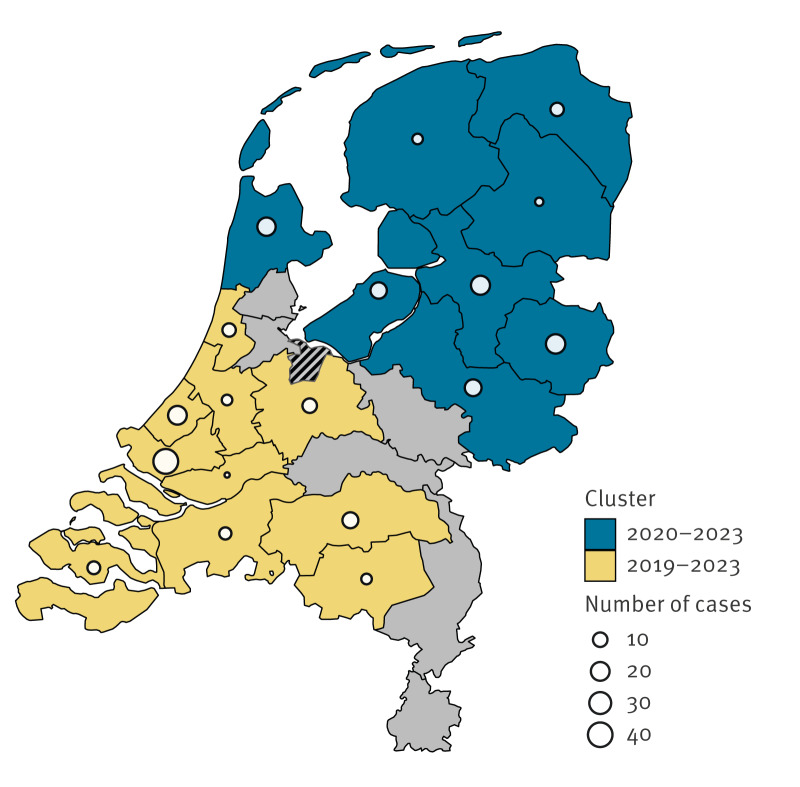
Spatiotemporal clustering of syphilis diagnoses among females and heterosexual males at sexual health centres in the Netherlands, 2011–2023 (n = 686)

Overall syphilis positivity differed between SHC regions. The Amsterdam region, in the west, had the lowest positivity (0.05%) and IJsselland, in the east, had the highest positivity (0.31%) (Figure 2). The global Moran’s I found global spatial autocorrelation (Moran’s I statistic: 0.37, p value: 0.002), indicating a clustering pattern. The SaTScan identified two significant spatiotemporal clusters (Figure 3). One spatiotemporal cluster was located in the south-west of the Netherlands and contained 10 SHC regions. It was significant for 2019–23, with a relative risk of 2.39 (p value < 0.001). The total number of syphilis diagnoses in the cluster was 114, with an expected number of 55.7 diagnoses. The other cluster was in the north of the country and included eight SHC regions. This cluster was significant for 2020–23, with a relative risk of 5.03 (p value < 0.001). The total number of syphilis diagnoses in the cluster was 105, with an expected number of 24.6 diagnoses.

There are 25 health regions in the country, of which 24 have a sexual health centre (SHC). The region Gooi and Vechtstreek (grey striped area) does not have an SHC, individuals from this region can be tested at SHCs in surrounding regions.

There are 25 health regions in the country, of which 24 have a sexual health centre (SHC). The region Gooi and Vechtstreek (grey striped area) does not have an SHC, and individuals from this region can be tested at SHCs in surrounding regions.

### Determinants of syphilis

All independent variables except sex work were significantly associated with syphilis in the univariate analysis (Table). HIV status showed the strongest correlation, with an odds ratio (OR) of 26.53 and 95% confidence interval (95% CI) of 18.05–37.51.

In the multivariable model, we found a significant association between all included independent variables and syphilis (Table). Living with HIV had the strongest association (adjusted OR (aOR): 17.48, 95% CI:  11.75–25.08). The model showed that the odds of having syphilis increased over time (aOR: 1.69, 95% CI: 1.40–2.05 for years 2015–19; aOR: 3.34, 95% CI: 2.62–4.23 for years 2020–21; aOR: 3.63, 95% CI: 2.92–4.50 for years 2022–23; compared with years 2011–14). Males were more likely to have syphilis than females (aOR: 2.24, 95% CI: 1.91–2.62), and individuals > 25 years were more likely to have syphilis than individuals aged ≤ 25 years (aOR: 2.37, 95% CI: 2.00–2.81). Other variables that showed a positive correlation with syphilis were having symptoms and having received a partner notification. A higher number of partners and being a Western migrant had a negative correlation with syphilis, while non-Western migrants were found to have higher odds of syphilis than those born in the Netherlands.

In the stratified analysis by age group, all determinants and directions of effects were the same as in the main analysis, except migration status. In the main analysis, Western migrants were less likely while non-Western migrants were more likely to have syphilis than those born in the Netherlands. In the sensitivity analysis conducted among individuals ≤ 25 years, migration status was no longer statistically significant. In the analysis including reporting an MSM partner, females with an MSM partner were found to have higher odds of syphilis than those without an MSM partner (aOR: 2.94, 95% CI: 1.76–4.64). In the analysis including partner notification for syphilis, heterosexuals with a syphilis notification were more likely to have syphilis than those without a syphilis notification (aOR: 93.93, 95% CI: 75.90–115.83).

## Discussion

Between 2011 and 2023, there were 686 syphilis diagnoses among females and heterosexual males attending Dutch sexual health centres. We found an increasing trend in syphilis positivity, from 0.05% in 2011 to 0.23% in 2023. Two ongoing space-time clusters were identified, in the south-west (2019–23) and in the north (2020–23) of the Netherlands. Living with HIV was found to be the strongest risk factor for syphilis.

The increasing trend in syphilis among heterosexuals between 2011 and 2023 was observed even after adjusting for changes in the population attending the SHC. However, the increase is still limited compared with the increase in EU/EEA reported by ECDC [4]. The increasing trend among females is concerning because of the risk of transmission during pregnancy. As in other European countries, the number of neonates and young infants with congenital syphilis has been low in the Netherlands, with 0–3 annual cases since 2014 [5,11]. While the Dutch pregnancy screening covers 99.99% of pregnant people, it is imperative to continue testing all pregnant people in order to prevent mother-to-child transmission [11]. Heterosexuals who fulfil the triage criteria have access to free STI testing at an SHC, while others have to be tested through their GP or via commercial testing, which is not free-of-charge. It has previously been found that in the Amsterdam region, GPs were responsible for 19% of the syphilis diagnoses among MSM, the remainder were diagnosed at the SHC [16]. It is unclear whether this is similar for heterosexuals. At a national level, the number of syphilis diagnoses at GPs has been reported to be too low for accurate incidence estimates, indicating that GPs do not play a major role in diagnosing syphilis among heterosexuals [11]. This could be due to low incidence among heterosexuals or because GPs are less likely to order syphilis tests. Data from commercial test providers are not available.

This study examines spatial syphilis trends among heterosexuals in the Netherlands. A previous study using SHC data on syphilis among MSM found clusters in the Amsterdam and Rotterdam regions, both major cities [17]. Our results indicate that the spatial pattern among heterosexuals is different; one spatiotemporal cluster included Rotterdam and some other urban areas, but the other cluster mainly included more rural areas with smaller SHCs. We accounted for testing criteria, such as symptoms and partner notification, but it is possible that some SHCs are stricter with triage for testing among heterosexuals and thus mainly test those with additional risk factors, resulting in high syphilis positivity in these regions. The clusters we found were large and spanned multiple years, indicating ongoing transmission and the introduction of new cases among heterosexuals over time.

Our results indicate that the Dutch testing criteria for heterosexuals target most of those individuals with a higher likelihood of syphilis. The testing criteria of being older than 25 years, having symptoms, and having received a partner notification were associated with higher odds of syphilis, especially syphilis-specific partner notification. This is consistent with a previous study on syphilis and HIV among heterosexuals older than 25 years using SHC data between 2015 and 2021 [18]. The current study, using a dataset with a longer time span (2011–23) and including individuals younger than 26 years, confirms these findings and strengthens the evidence for these determinants of a syphilis diagnosis.

Many previous studies have shown that MSM living with HIV have an increased risk of syphilis [1]. Evidence for this association among heterosexuals is more limited, with a few studies showing a similar association [19,20]. Despite the low number of heterosexuals living with HIV in our dataset, we found that living with HIV increased the odds of syphilis for heterosexuals. In the Netherlands, it is estimated that 94% of women living with HIV are in care and, of those, 95% are on antiretroviral therapy (ART); for heterosexual men, this is 89% and 93%, respectively [11]. In the Netherlands, living with HIV is not a triage indication for access to free testing at the SHC, and it is also not one of the indications for a syphilis test [14]. There is no specific guideline for syphilis testing at HIV clinics; the testing guideline advises bacterial STI testing based on risk behaviour at the first consultation and gives no recommendation for subsequent check-ups [21]. Due to the increased odds of syphilis infection, it is recommended that heterosexuals living with HIV receive a standard syphilis test, either at check-ups at HIV clinics or when visiting the SHC. Further research is needed to verify whether routine syphilis testing among heterosexuals would be cost-effective.

The odds of syphilis were higher for non-Western migrants than for heterosexuals born in the Netherlands. Migrants receive a standard syphilis test on their first consultation at an SHC, regardless of their age or other testing indications. Syphilis testing in further consultations depends on their risk behaviour. We found that 21% of syphilis diagnoses were among non-Western migrants in their first consultation at a SHC, indicating that this testing indication is effective in identifying syphilis in this group.

We found that individuals with more than one sexual partner had lower odds of syphilis than those with one or no partner in the past 6 months. This is not consistent with previous studies, which found that females with multiple sexual partners had a higher likelihood of syphilis than females with one partner [22,23]. Our findings could be related to testing behaviour; individuals with more partners might test more often, while those with a stable partner might estimate their risk of STI as low, and only get tested if their partner had an STI or if they themselves have symptoms. However, when adjusting for partner notification and symptoms, the odds for individuals with more than one partner were still lower than for those with zero or one partner. We did not have information on whether sexual partners were stable or casual, or whether sexual contacts were monogamous, and were therefore unable to adjust for this.

We found that females with an MSM partner had higher odds of syphilis than females without an MSM partner. This indicates potential bridging of syphilis from the MSM sexual network, where syphilis is more common and positivity has increased in the past 10 years [11]. Previous studies in England and Australia have found genomic clusters across MSM and heterosexual networks, suggesting that there is syphilis transmission between these groups [6,7]. Genomic epidemiological analysis of syphilis among both heterosexuals and MSM is necessary to confirm bridging in the Netherlands.

Our study had several limitations. Firstly, results are not generalisable to the entire Dutch heterosexual population, because SHCs only provide STI testing for specific risk groups. The positivity rates in this study are therefore an overestimation of the syphilis incidence in the general population. Individuals who do not fulfil the triage requirements are advised to get tested at their GP or to access commercial testing. It was not possible to link the SHC data to data from other sources. Secondly, criteria for syphilis testing changed over time, particularly for individuals aged 25 years or younger. We accounted for this by adding calendar year as a confounder in the multivariate model. Sensitivity analysis showed that findings were mostly consistent between the age groups, except that migration status was no longer a significant determinant for individuals aged 25 or younger. Thirdly, we did not have data on genomic types or contact tracing, therefore it was not possible to determine whether the space-time clusters were epidemiologically or microbiologically linked. Fourthly, a person identifier was not available for all years, therefore it was only possible to link multiple consultations from the same person for part of the study period. Fifthly, there may be social-desirability bias, as sexual behaviour was self-reported. This may have led to misclassification of some MSM as heterosexual men. However, this risk is minimised as consultations at SHC are undertaken by nurses who are specialised in sexual health and collecting sensitive personal information. 

## Conclusion

This study shows there is a need for increased awareness of syphilis among key heterosexual populations in the Netherlands. While the number of diagnoses is low, positivity among heterosexuals has increased significantly since 2011. Syphilis was found in all regions of the Netherlands, with significantly higher positivity rates in recent years in the west and north-east of the country. Current syphilis testing among groups at increased risk, such as migrants and those with symptoms or a partner notification, should continue at SHCs. Since living with HIV was the strongest risk factor for syphilis, increased testing among heterosexuals living with HIV, both at SHCs and HIV treatment clinics, is recommended.

## Data Availability

Data are available upon reasonable request. This study uses data from the Dutch national registration of sexual health centre consultations (SOAP). Data can be requested for scientific use from the SOAP registration committee (contact: soap@rivm.nl).
